# Corrigendum

**DOI:** 10.1002/ccr3.6259

**Published:** 2022-09-16

In the article entitled “Intraoperative recurrent laryngeal nerve monitoring in unconventional thyroid surgery” which was previously published incorrectly in Volume 10 Issue 7 of *Clinical Case Reports*, all except the fifth author's first and last names were wrongly swapped. The names in the citation were also published incorrectly

The names should be as follows: Filippo Carta, Valeria Marrosu, Valeria Pinto, Melania Tatti, Mauro Bontempi, Cinzia Mariani, Roberto Puxeddu

Citation should be:

Carta F, Marrosu V, Pinto V, et al. Intraoperative recurrent laryngeal nerve monitoring in unconventional thyroid surgery. Clin Case Rep. 2022;10:e06137. doi:10.1002/ccr3.6137

The authors apologize for the above errors.
